# Prolonged use of human insulin increases breast cancer risk in Taiwanese women with type 2 diabetes

**DOI:** 10.1186/s12885-015-1876-7

**Published:** 2015-11-04

**Authors:** Chin-Hsiao Tseng

**Affiliations:** 1 Department of Internal Medicine, National Taiwan University College of Medicine, No. 7 Chung-Shan South Road, Taipei, 100 Taiwan; 2 Division of Endocrinology and Metabolism, Department of Internal Medicine, National Taiwan University Hospital, Taipei, Taiwan

**Keywords:** Breast cancer, Human insulin, Type 2 diabetes mellitus, Incidence, Taiwan

## Abstract

**Background:**

Human insulin is commonly used to treat hyperglycemia in patients with diabetes, but its potential link with female breast cancer is under debate. This study investigated whether human insulin use might be associated with breast cancer risk in Taiwanese women with type 2 diabetes.

**Methods:**

The reimbursement databases of all Taiwanese diabetic patients from 1996 to 2009 were retrieved from the National Health Insurance. An entry date was set at 1 January 2004 and a total of 482,033 women with type 2 diabetes were followed up for breast cancer incidence until the end of 2009. Incidences for ever-users, never-users and subgroups of human insulin exposure (using tertile cutoffs of time since starting insulin, cumulative dose and cumulative duration of insulin) were calculated and the adjusted hazard ratios were estimated by Cox regression. The potential risk modification by concomitant treatment with metformin, statin and angiotensin converting enzyme inhibitor/angiotensin receptor blocker (ACEI/ARB) was also evaluated.

**Results:**

There were 59,798 ever-users and 422,235 never-users of human insulin, with respective numbers of incident breast cancer of 559 (0.93 %) and 4,711 (1.12 %), and respective incidence of 207.9 and 215.1 per 100,000 person-years. The overall adjusted hazard ratio (95 % confidence interval) did not show a significant association with insulin [1.033 (0.936-1.139)]. However, patients in the third tertiles of dose–response parameters might show a significantly higher risk of breast cancer while compared to never-users: 1.185 (1.026-1.368), 1.260 (1.096-1.450) and 1.257 (1.094-1.446) for ≥67 months for time since starting insulin, ≥39,000 units for cumulative dose of insulin, and ≥21.8 months for cumulative duration of insulin, respectively. Additional analyses suggested that the breast cancer risk associated with human insulin use might be beneficially modified by concomitant use of metformin, statin and ACEI/ARB.

**Conclusions:**

This study discloses a significantly higher risk of breast cancer associated with prolonged use of human insulin. The increased risk of breast cancer associated with human insulin use may be modified by medications such as metformin, statin and ACEI/ARB.

## Background

Diabetes mellitus may increase the risk of breast cancer in terms of incidence and mortality [1, 2]. However, whether insulin use can be responsible for the increased risk of diabetes-related breast cancer is still under debate. Some *in vitro* and observational studies provide evidence for a potential link between insulin and breast cancer. For examples, insulin can be found in human breast cancer tissues [3], and some breast cancers are responsive to insulin and administration of insulin/insulin-like growth factor 1 (IGF-1) receptor family kinase inhibitor or alloxan to produce a status of hypoinsulinemia by destroying the insulin producing pancreatic β-cells may inhibit tumor growth of breast cancer cell line [4]. Mammalian target of rapamycin (mTOR) is activated by insulin and insulin-mediated breast cancer progression in patients with type 2 diabetes mellitus may be abrogated by inhibition of mTOR [5]. The Women’s Health Initiative study prospectively showed that elevated insulin level may predict postmenopausal breast cancer [6].

Insulin glargine, a long-acting insulin analog, has a 6- to 8-fold higher binding affinity to IGF-1 receptor than human insulin [7]. Insulin glargine may stimulate the proliferation of breast cancer cell lines [8, 9]. A recent nested case–control study suggested that insulin glargine may increase the risk of all cancer, while human insulin and other types of insulin analogs do not increase cancer risk [10]. Long-term use or high dose of insulin glargine may significantly increase the risk of breast cancer [11–14]. Therefore insulin glargine may have different effects on cancer development compared to other forms of insulin.

Insulin glargine was the first insulin analog introduced into the market of Taiwan in February 2004, but human insulin remains the most commonly used insulin in clinical practice. Therefore, it is clinically important to clarify whether human insulin can be associated with breast cancer. The purpose of the present study was to evaluate whether human insulin use without confounding exposure to insulin analogs would increase the risk of breast cancer in female patients with type 2 diabetes mellitus, by using the National Health Insurance (NHI) databases of Taiwan. The reasons for precluding the investigation of the effect of insulin glargine in the present study is due to the observation in clinical practice that most users of insulin might have been prescribed human insulin for a while before being given insulin glargine or might have been treated with a combination of human insulin and insulin analogs, making it difficult to dissect an effect of insulin glargine independent of human insulin. Additionally, the shorter duration of exposure to insulin glargine in most patients and the small number of cases prescribed insulin glargine during the study period would lead to a lack of sufficient statistical power for subgroup analyses in the study.

## Methods

This is a nationwide cohort analysis using the NHI databases including all patients with a diagnosis of diabetes mellitus during the period from 1996 to 2009 in Taiwan. The study was approved by an ethic review board of the National Health Research Institutes with registered approval number 99274. Written informed consent from the participants was not required according to local regulations because the identification information of the individuals was scrambled and de-identified prior to analysis for the protection of privacy.

Since March 1995 a compulsory and universal system of health insurance (the so-called NHI) was implemented in Taiwan. All contracted medical institutes must submit computerized and standard claim documents for reimbursement. More than 99 % of citizens are enrolled in the NHI, and >98 % of the hospitals nationwide are under contract with the NHI. The average number of annual physician visits in Taiwan is one of the highest around the world, at approximately 15 visits per year per capita in 2009.

The National Health Research Institutes is the only organization approved, as per local regulations, for handling the NHI reimbursement databases for academic research. The databases contain detailed records on every visit for each patient, including outpatient visits, emergency department visits and hospital admission. The databases also include principal and secondary diagnostic codes, prescription orders, and claimed expenses.

The identification information of the individuals was de-identified for the protection of privacy. Diabetes was coded 250.XX and breast cancer 174, based on the *International Classification of Diseases, Ninth Revision, Clinical Modification* (ICD-9-CM).

The entry date was set on 1 January 2004. The selection of this entry date was due to the fact that insulin glargine or other insulin analogs were not available before this date in Taiwan. Therefore at the time of enrollment, all patients would not have been exposed to insulin glargine or other insulin analogs. Patients who might happen to be prescribed insulin glargine or other insulin analogs after the entry date were censored on the date of their prescriptions as mentioned later in the calculation of person-years of follow-up. These procedures assured that none of the patients were exposed to insulin glargine or other insulin analogs during follow-up in either the human insulin ever-users or never-users defined in the study.

We first retrieved the databases of all patients who had been diagnosed as having diabetes and under treatment with either oral antidiabetic drugs (OAD) or insulin during the period of 1996–2009 from the whole nation and who remained in the insurance program after the entry date (*n* = 1,557,468). After excluding men (*n* = 930,327), patients who had a diagnosis of diabetes after the year 2004 (*n* = 534,522), patients who held a Severe Morbidity Card as having type 1 diabetes (*n* = 5,894, in Taiwan, patients with type 1 diabetes were issued a so-called “Severe Morbidity Card” after certified diagnosis and they were waived for much of the co-payments), patients having a diagnosis of breast cancer before 2004 (*n* = 11,969), those who died (*n* = 62,176) or withdrew from the NHI (*n* = 9,512) before entry date, duplicated identification number (*n* = 106), unclear information on date of birth or sex (*n* = 5,122), and patients who had been prescribed insulin only once (*n* = 70,151, some patients might have been given insulin temporarily for certain medical conditions but they might not be real cases of diabetes), a total of 482,065 female patients with a diagnosis of type 2 diabetes mellitus and under therapy with OAD or insulin were recruited. A total of 32 patients who had been prescribed insulin during the first year (i.e., 1996) of the availability of NHI database were further excluded to assure the accuracy in the calculation of the dose–response parameters of insulin exposure as described below.

Those who had ever been prescribed insulin before entry date were defined as ever-users (*n* = 59,798, 12.4 %); and never-users (*n* = 422,235, 87.6 %) were defined as those who had never been prescribed insulin before entry date. To evaluate whether a dose–response relationship could be seen between human insulin and breast cancer, tertile cutoffs for the following three variables were used: time since starting human insulin in months, cumulative duration of human insulin therapy in months and cumulative dose of human insulin in units, were calculated from the databases and used for analyses [15, 16].

All comorbidities and covariates were determined as a status/diagnosis before the entry date. The ICD-9-CM codes for the comorbidities were [17]: obesity 278, nephropathy 580–589, hypertension 401–405, chronic obstructive pulmonary disease (a surrogate for smoking) 490–496, cerebrovascular disease 430–438, ischemic heart disease 410–414, peripheral arterial disease 250.7, 785.4, 443.81 and 440–448, eye disease 250.5, 362.0, 369, 366.41 and 365.44, dyslipidemia 272.0-272.4, congestive heart failure 398.91, 402.11, 402.91, 404.11, 404.13, 404.91, 404.93 and 428, and cancer other than breast cancer 140–208 (excluding 174). Medications included pioglitazone, rosiglitazone, sulfonylurea, meglitinide, metformin, acarbose, statin, fibrate, angiotensin converting enzyme inhibitor and/or angiotensin receptor blocker (ACEI/ARB), calcium channel blocker, non-steroidal anti-inflammatory drugs and estrogen. Baseline characteristics between ever-users and never-users of human insulin were compared by Student’s t test for age and diabetes duration and by Chi-square test for other variables.

The incidence density of breast cancer was calculated for ever-users and never-users and for different subgroups of exposure. The numerator for the incidence was the number of patients with incident breast cancer during the 6-year follow-up (from 1 January 2004 to 31 December 2009), and the denominator was the person-years of follow-up. For ever-users, the follow-up duration was either censored at the date of initiation of insulin glargine or other insulin analogs, or breast cancer diagnosis or at the date of the last record of the available reimbursement databases in individuals without incident breast cancer. For never-users, the follow-up was censored at the date of insulin initiation (including human insulin or insulin analogs) or breast cancer diagnosis or the last reimbursement record, depending on whichever occurring first. This ensured no exposure to insulin of any form throughout the whole follow-up period until censor in the referent group of never-users; and no exposure to insulin glargine or other insulin analogs in the group of ever-users of human insulin.

Cox proportional hazards regression was performed to estimate the hazard ratios for breast cancer for ever-users versus never-users, and for the various subgroups of dose–response parameters based on the tertile cutoffs using never-users of human insulin as the referent group. The models were adjusted for all variables compared previously as baseline characteristics between ever-users and never-users (primary model). In all regression models, age and diabetes duration (logarithmically transformed to normalize the data) were treated as continuous variables.

To examine whether the results might be consistent under different conditions, the following models were conducted as sensitivity analyses: 1) adjusting only for the following important risk factors of breast cancer: age, diabetes duration, obesity, estrogen, metformin, statin and ACEI/ARB (sensitivity model I); and 2) including human insulin exposure post entry date but prior to breast cancer diagnosis in the calculation of the dose–response parameters.

Some medications commonly used in patients with diabetes such as metformin [18, 19], statin [20] and ACEI/ARB [21, 22] may affect the risk of breast cancer. In order to evaluate the potential risk modification by these medications with short- or long-term exposure, additional Cox regression models were created by categorizing the patients into various groups of exposure to insulin and to these medications for <2 years or ≥ 2 years. In consideration that some medications might be used after the entry date, the exposure duration to insulin and the other medications were calculated until censor.

Analyses were conducted using SAS statistical software, version 9.3 (SAS Institute, Cary, NC). *P* < 0.05 was considered statistically significant.

## Results

Table 1 compares the baseline characteristics between ever-users (*n* = 59,798) and never-users (*n* = 422,235) of human insulin. All variables differed significantly between the two groups. Ever-users are characterized by older age, longer diabetes duration, higher proportions of all comorbidities and other cancer, and higher proportions of using other medications.

**Table 1:** Baseline characteristics between never-users and ever-users of human insulin

	Human insulin				
Variables	Never-users		Ever-users		*P* value
	*n*	%	*n*	%	
*n* = 482033	422235		59798		
Age (years)^a^	62.54 ± 12.22		63.70 ± 12.65		<0.0001
Diabetes duration (years)^a^	5.12 ± 2.52		7.12 ± 1.65		<0.0001
Obesity	6082	1.44	974	1.63	0.0003
Nephropathy	39290	9.31	19533	32.66	<0.0001
Hypertension	228224	54.05	46172	77.21	<0.0001
Chronic obstructive pulmonary disease	51257	12.14	16773	28.05	<0.0001
Cerebrovascular disease	51910	12.29	19239	32.17	<0.0001
Ischemic heart disease	86668	20.53	25673	42.93	<0.0001
Peripheral arterial disease	41898	9.92	17216	28.79	<0.0001
Eye disease	48952	11.59	17588	29.41	<0.0001
Dyslipidemia	182015	43.11	33585	56.16	<0.0001
Congestive heart failure	24242	5.74	11009	18.41	<0.0001
Other cancer prior to baseline	39359	9.32	7672	12.83	<0.0001
Pioglitazone	9581	2.27	3503	5.86	<0.0001
Rosiglitazone	38135	9.03	16488	27.57	<0.0001
Sulfonylurea	304121	72.03	54487	91.12	<0.0001
Meglitinide	27501	6.51	11588	19.38	<0.0001
Metformin	259203	61.39	53420	89.33	<0.0001
Acarbose	33979	8.05	14763	24.69	<0.0001
Statin	97657	23.13	23400	39.13	<0.0001
Fibrate	77010	18.24	19220	32.14	<0.0001
Angiotensin converting enzyme inhibitor/angiotensin receptor blocker	169738	40.20	41504	69.41	<0.0001
Calcium channel blocker	133391	31.59	34541	57.76	<0.0001
Non-steroidal anti-inflammatory drugs	286403	67.83	56369	94.27	<0.0001
Estrogen	37775	8.95	8583	14.35	<0.0001

^a^Age and diabetes duration are expressed as mean ± standard deviation

Table 2 shows the incidences of breast cancer between ever-users and never-users of human insulin, and among the different tertiles of the dose–response parameters of human insulin exposure. In the primary model, the overall hazard ratio (1.033, 95 % confidence interval: 0.936-1.139) for ever-users versus never-users was not statistically significant, but a significantly higher risk was observed in the third tertiles of all three dose–response parameters. Similar findings were noted in sensitivity model I where only a subset of important risk factors of breast cancer was adjusted for. Additionally, a significantly lower risk of breast cancer was observed for the first tertiles of cumulative dose and cumulative duration of exposure. In sensitivity model II where human insulin exposure post entry date but prior to breast cancer diagnosis was also included in the calculation of the dose–response parameters, significantly higher risk of breast cancer was still observed in the third tertiles of time since starting human insulin and cumulative duration of human insulin exposure, and in the second tertile of cumulative dose of human insulin.

**Table 2:** Exposure to human insulin and incidences of breast cancer and the adjusted hazard ratios comparing exposed to unexposed

Exposure to human insulin	Case number	Incident breast cancer	%	Person-years	Incidence rate (per 100,000 person-years)	Primary model			Sensitivity model I			Sensitivity model II		
						HR	95 % CI	*P*	HR	95 % CI	*P*	HR	95 % CI	*P*
Never-users	422235	4711	1.12	2190014.8	215.1	1.000			1.000			1.000		
Ever-users	59798	559	0.93	268941.3	207.9	1.033	(0.936-1.139)	0.5200	0.994	(0.907-1.090)	0.9013	1.033	(0.936-1.139)	0.5200
Time since starting human insulin (months)														
Never-users	422235	4711	1.12	2190014.8	215.1	1.000			1.000			1.000		
<35	19409	165	0.85	88579.0	186.3	0.924	(0.787-1.085)	0.3358	0.877	(0.750-1.027)	0.1024	0.924	(0.787-1.085)	0.3358
35-67	19817	173	0.87	89856.1	192.5	0.988	(0.843-1.158)	0.8813	0.944	(0.809-1.101)	0.4603	0.988	(0.843-1.158)	0.8813
≥67	20572	221	1.07	90506.3	244.2	1.185	(1.026-1.368)	0.0209	1.161	(1.011-1.333)	0.0342	1.185	(1.026-1.368)	0.0209
Cumulative dose of human insulin exposure (units)														
Never-users	422235	4711	1.12	2190014.8	215.1	1.000			1.000			1.000		
<4,000	19106	151	0.79	89076.3	169.5	0.872	(0.738-1.030)	0.1069	0.819	(0.695-0.965)	0.0169	0.982	(0.837-1.152)	0.8264
4,000-39,000	20322	170	0.84	89166.3	190.7	0.960	(0.818-1.126)	0.6148	0.919	(0.787-1.073)	0.2863	1.184	(1.017-1.380)	0.0298
≥39,000	20370	238	1.17	90698.7	262.4	1.260	(1.096-1.450)	0.0012	1.239	(1.084-1.416)	0.0017	0.961	(0.828-1.115)	0.5987
Cumulative duration of human insulin exposure (months)														
Never-users	422235	4711	1.12	2190014.8	215.1	1.000			1.000			1.000		
<2.8	19751	151	0.76	90689.0	166.5	0.855	(0.724-1.009)	0.0643	0.809	(0.687-0.953)	0.0112	0.855	(0.724-1.009)	0.0643
2.8-21.7	19716	167	0.85	87633.9	190.67	0.981	(0.834-1.153)	0.8142	0.927	(0.792-1.084)	0.3407	0.981	(0.834-1.153)	0.8142
≥21.8	20331	241	1.19	90618.4	266.0	1.257	(1.094-1.446)	0.0013	1.240	(1.085-1.416)	0.0015	1.257	(1.094-1.446)	0.0013

HR: hazard ratio, CI: confidence interval

Primary model: adjusted for all variables listed in Table 1.

Sensitivity model I: adjusting only for important risk factors of breast cancer including age, diabetes duration, obesity, estrogen, metformin, statin and angiotensin converting enzyme inhibitor/angiotensin receptor blocker.

Sensitivity model II: including human insulin exposure post entry date but prior to breast cancer diagnosis in the calculation of the dose–response parameters.

Table 3 shows the results of the models that considered the potential risk modification by the use of other medications. In Model I, patients treated with human insulin alone without any OAD had a significantly higher risk of breast cancer (hazard ratio: 1.413, 95 % confidence interval: 1.030-1.940). On the other hand, patients treated with OAD with a short duration of insulin treatment for <2 years had a significantly lower risk, while the risk became neutral in those who had been treated with OAD plus human insulin for ≥2 years. Model II suggested that human insulin users who also had been treated with metformin for ≥2 years had a significantly lower risk of breast cancer (hazard ratio: 0.798, 95 % confidence interval: 0.741-0.859). Model III suggested that human insulin users who also had been treated with statin for as short as <2 years had a significantly lower risk of breast cancer. The beneficial effect seemed to persist in patients who had used statin for a longer duration of ≥2 years. In Model IV, human insulin users without use of ACEI/ARB or with a short-term use of ACEI/ARB for <2 years had a significantly lower risk of breast cancer, while those who had been treated with ACEI/ARB for a longer term (≥2 years) showed a neutral risk association.

**Table 3:** Models considering the potential risk modification on the link between human insulin and breast cancer by other medications commonly used in patients with type 2 diabetes mellitus

Model	*n*/*N*	HR	95 % CI	*P*
Model I				
Never users of insulin	4165/345654	1.000		
Users of human insulin alone without any OAD	39/2939	1.413	(1.030-1.940)	0.0323
OAD plus human insulin < 2 years	636/88019	0.728	(0.668-0.793)	<0.0001
OAD plus human insulin ≥ 2 years	430/45421	0.983	(0.882-1.096)	0.7587
Model II				
Never users of insulin	4165/345654	1.000		
Users of human insulin alone or plus OAD excluding metformin	82/7827	1.127	(0.904-1.405)	0.2893
Users of human insulin plus metformin < 2 years	23/2285	0.782	(0.519-1.178)	0.2398
Users of human insulin plus metformin ≥ 2 years	1000/126267	0.798	(0.741-0.859)	<0.0001
Model III				
Never users of insulin	4165/345654	1.000		
Users of human insulin plus without use of statin	442/51396	0.928	(0.839-1.027)	0.1484
Users of human insulin plus statin < 2 years	63/8601	0.654	(0.509-0.840)	0.0009
Users of human insulin plus statin ≥ 2 years	600/76382	0.789	(0.718-0.866)	<0.0001
Model IV				
Never users of insulin	4165/345654	1.000		
Users of human insulin plus without use of ACEI/ARB	223/26367	0.822	(0.716-0.943)	0.0052
Users of human insulin plus ACEI/ARB < 2 years	492/69952	0.745	(0.675-0.822)	<0.0001
Users of human insulin plus ACEI/ARB ≥ 2 years	390/40060	1.005	(0.895-1.128)	0.9370

*n*: case number of incident breast cancer, *N*: case number followed

HR: hazard ratio, CI: confidence interval

OAD: oral antidiabetic drug, ACEI: angiotensin converting enzyme inhibitor, ARB: angiotensin receptor blocker

## Discussion

The findings of this large population-based study suggested that prolonged exposure to human insulin may increase the risk of breast cancer after multivariable adjustment (Table 2). Significantly higher risk of approximately 25 % can be consistently observed in the third tertile of a cumulative duration of human insulin exposure ≥21.8 months in all three models including the primary model and the two sensitivity models (Table 2). The increased risk can be as high as 40 % in a subset of patients who had been treated with insulin alone without the use of any OAD (Model I, Table 3). Additional analyses suggested that the risk of breast cancer associated with human insulin use might be modified by a combination use of other medications including metformin, statin and ACEI/ARB (Models II to IV, Table 3).

Insulin per se may be able to increase the risk of breast cancer through several mechanisms. First, insulin is a well-known growth factor, which may activate the proliferation of breast cancer cells through its interaction with the insulin receptor or the IGF-1 receptor [23] and through activating the mTOR pathway [5]. Second, exogenous insulin administration is always followed by increased body weight, which is a key feature leading to insulin resistance, hyperinsulinemia, hyperglycemia, increased oxidative stress and proinflammation. All of these can contribute to the increased risk of various types of cancer in epidemiological studies [24–27]. Third, clinically it is not easy to obtain optimal physiological level of insulin to sustain glycemic control by exogenous insulin administration and therefore hyperinsulinemia is unavoidable in the presence of insulin resistance, leading to a vicious cycle favoring the development of breast cancer.

Diabetes severity may be a prime driver of breast cancer risk, and insulin is always used at a late stage of diabetes when pancreatic β cells are exhausted and most OAD fail to adequately control blood glucose. Therefore, indication bias may exist when insulin is used in patients having more comorbidities and using more concomitant drugs that may also affect the risk of breast cancer. In the present study, although most comorbidities and medications that may be related to the exposure to human insulin and/or to breast cancer have been considered as potential confounders (Tables 1, 2 and 3), it was not able to neatly segregate the confounding effect of other indicators of diabetes severity such as glycemic control, which might also be highly correlated with the use of insulin, from the effect of insulin per se. All of the indicators of disease severity may also progress in intensity with respect to the length of diabetes duration and thus to the duration of insulin exposure. Therefore, a link between the dose–response parameters of insulin use might also reflect a link with progressive pathophysiological changes associated with increasing diabetes duration such as insulin resistance, inflammation, oxidative stress, and aggravated hyperglycemia. It is true that diabetes per se may increase the risk of breast cancer, disregarding the use of insulin, as observed in a recent Danish study [28], suggesting a possible link through some underlying features of diabetes and not through exogenous insulin administration. However this Danish study fell short of a lack of dose–response analysis, being unable to differentiate the use of human insulin and insulin glargine, and a lack of adjustment for potential confounders. Actually if we did not consider the dose–response analyses, the overall hazard ratio was not significant (Table 2).

Some studies suggested that patients using insulin glargine may have a higher risk of cancer than patients using human insulin [10]. However, a recent analysis including 31 randomized clinical trials did not find a higher incidence of cancer, including breast cancer, in patients using insulin glargine in comparison to comparators [29]. In the present study, the possibility of exposure to insulin glargine or other insulin analogs had been excluded in the calculation of the person-years during the follow-up. Therefore, whether insulin glargine may increase breast cancer risk in our population is an issue awaiting further confirmation.

Taken together, although the present study demonstrated a link between prolonged use of human insulin and breast cancer risk, it was not able to clearly discern the cause-effect relationship due to the inherent bias across the two groups of patients featuring a potential direct impact of diabetes severity on breast cancer risk. However, because of the biological plausibility of an insulin effect on breast cancer development and the relationship featuring a lack of association during the initial period of insulin use and the requirement of an adequate incubation period for a significant risk (Table 2), a carcinogenic effect of prolonged use of human insulin on breast tissue could not be completely excluded. The finding of a significant link between insulin use for 3 or more years and mortality from breast cancer in a recent follow-up of a large cohort of female patients in Taiwan [24] adequately reflects a close link between insulin use for approximately 2 years and breast cancer risk (Table 2) as shown in the present study.

Metformin is commonly used among the studied patients (Table 1) and it has been shown to reduce the risk of breast cancer in previous studies [18, 19]. This beneficial effect of metformin could also be demonstrated in the present study (Model II, Table 3). It is clearly demonstrated here that patients who had used only human insulin without the use of any other OAD might suffer from a significantly higher risk of breast cancer with an estimated hazard ratio of 1.413 (95 % confidence interval: 1.030-1.940), while those who had used OAD plus a short-term human insulin use of <2 years might have a significantly lower risk of breast cancer (Model I, Table 3). However, when human insulin had been used for a prolonged duration of ≥ 2 years in addition to OAD, the lower risk observed in the previous group attenuated and became neutral (Model I, Table 3). The lower risk associated with OAD when human insulin had been used for <2 years (Model I, Table 3) might reflect a beneficial effect of metformin (Model II, Table 3), which was a commonly used OAD (Table 1).

The analysis in Model III of Table 3 also suggested a beneficial effect of statin after a short duration of its use for <2 years, which persisted after a prolonged duration of ≥2 years. Hypercholesterolemia may accelerate breast tumor growth in mice [30], probably through the interaction between 27-hydroxycholesterol (a primary metabolite of cholesterol) and the estrogen receptor and liver X receptor [31]. A recent UK study also suggested that hypercholesterolemia may possibly increase the risk of breast cancer in humans [32]. Therefore, it is not known whether the lower risk associated with statin use could be due to the biological effects of statin per se or due to an effective reduction in serum cholesterol level associated with statin use. It is worthy to point out that even though highly lipophilic statin, such as simvastatin, but not hydrophilic statin, has been shown to reduce breast cancer risk [33], there is a debate with regards to an increased risk of breast cancer associated with statin use, especially among the elderly [34].

The association between ACEI/ARB and breast cancer risk has been controversial. While some studies suggested a lack of association [35, 36], a study conducted in Taiwan suggested a lower risk of breast cancer associated with the use of ACEI [37] and another study conducted in Turkey suggested that patients with breast cancer who used ACEI/ARB might have a lower risk of recurrence and disease progression [38]. On the other hand, studies conducted in Taiwan [36] and in the USA [39] suggested a likely risk association with another class of antihypertensive drugs, the calcium channel blockers. ACEI/ARB are considered the first-line antihypertensive treatment in patients with diabetes, but a combination therapy with a calcium channel blocker, which is more potent in blood pressure lowering, is always required in diabetic patients with long-term hypertension. Taking into consideration the algorithm of the use of antihypertensive agents in the diabetic patients, patients not using ACEI/ARB in the present study might mainly represent those without hypertension and patients who had been using ACEI/ARB for <2 years might represent those with hypertension for a short period of time. On the other hand, diabetic patients who had been treated with ACEI/ARB for 2 or more years might have represented those with a long-term hypertension in whom a combination therapy with a calcium channel blocker is always necessary. Therefore, the attenuation of the beneficial effect of ACEI/ARB in patients who had been using these agents for ≥2 years (Model IV, Table 3) might also be explained by the effect of other antihypertensive agents added on top of ACEI/ARB such as calcium channel blockers. It should be admitted that the present study was not aimed at evaluating the risk of breast cancer associated with antihypertensive treatment and therefore further studies are necessary to clarify the risk association of breast cancer with the various classes of antihypertensive agents.

Obesity, reproductive factors and hormone use have been identified as risk factors of breast cancer [40–42]. In the present study a diagnosis of obesity and the use of estrogen had been included as potential confounders, but information of reproductive factors was not available in the NHI database. It should be pointed out that the prevalence of obesity was much underestimated in either the ever-users or never-users of insulin by using a diagnosis of obesity as a surrogate (Table 1). In a previous epidemiologic survey, the prevalence of obesity in patients with diabetes was 33.5 % and 7.1 %, respectively, by using a body mass index cutoff of ≥25 and ≥30 kg/m^2^ [43]. Therefore, a residual confounding of obesity could not be excluded in the present study. The lack of adjustment for reproductive factors might probably exert negligible confounding effect because a confounder needs to be associated with both exposure and disease, and should not be an intermediate variable in the causal pathway [44]. There is probably no evidence to suggest a link between reproductive factors and insulin use.

This study has several strengths. The databases included all claim records on outpatient visits, emergency department visits and hospital admission, and we caught the diagnoses from all sources. Cancer is considered a severe morbidity by the NHI and most medical co-payments can be waived. Furthermore, there is a low drug cost-sharing required by the NHI and patients with certain conditions such as low-income household, veterans or patients with prescription refills for chronic disease are exempted from the drug cost-sharing [45]. Therefore the detection rate of breast cancer would not tend to differ among different social classes. The use of medical record also reduced the potential bias related to self-reporting. Furthermore, we excluded patients with type 1 diabetes mellitus to demonstrate a link with type 2 diabetes mellitus; and excluded the potential contamination of the use of insulin glargine or other insulin analogs to demonstrate a link with human insulin. Because the databases were derived from the whole population, another important strength was an exclusion of potential selection bias related to sampling error.

Study limitations included a lack of actual measurement data of confounders such as obesity, smoking, alcohol drinking, family history, lifestyle, diet, reproductive factors and genetic parameters. In addition, we did not have biochemical data such as blood glucose level, insulin, or C-peptide for evaluating their impact. Finally, the present study was not able to evaluate the histological patterns, molecular markers (such as the expression of estrogen receptor) or clinical stages of breast cancer. According to the Taiwan Cancer Registry, 88.7 % of all cases with breast cancer may have invasive ductal carcinoma [46]. Therefore the breast cancer in the present study might reasonably be related to this histological type. Although misclassification of breast cancer might occur, such a probability was low because labeled diagnoses should be printed out in all prescriptions handed to the patients. Mislabeling of a cancer diagnosis would not be acceptable to the patients when they saw the diagnosis.

## Conclusions

The present study reveals a significantly higher risk of breast cancer associated with prolonged use of human insulin in female patients with type 2 diabetes mellitus. Such a risk can be consistently demonstrated when cumulative duration of human insulin exposure is ≥21.8 months. However, whether such an effect is independent of disease severity, which is highly correlated with the use of insulin, remains to be clarified. The link between human insulin can also be beneficially modified by a combination therapy with metformin, statin or ACEI/ARB. Whether insulin glargine or other insulin analogs may increase the risk of breast cancer is not evaluated in the present study, but this can be an important issue worthy of further investigation.

## Abbreviations

ACEI: angiotensin converting enzyme inhibitor
ARB: angiotensin receptor blocker
ICD-9-CM: International Classification of Diseases, Ninth Revision, Clinical Modification
IGF-1: insulin-like growth factor 1
mTOR: mammalian target of rapamycin
NHI: National Health Insurance
OAD: oral antidiabetic drug
